# Identification of fibrinogen as a natural inhibitor of MMP-2

**DOI:** 10.1038/s41598-019-40983-y

**Published:** 2019-03-13

**Authors:** Hassan Sarker, Eugenio Hardy, Ayman Haimour, Walter P. Maksymowych, Lorenzo D. Botto, Carlos Fernandez-Patron

**Affiliations:** 1 Department of Biochemistry, Faculty of Medicine and Dentistry, University of Alberta, Edmonton, AB T6G 2H7 Canada; 2 Biotechnology Laboratory, Study Center for Research and Biological Evaluations, Institute of Pharmacy and Foods, University of Havana, Havana, P.O. Box 13600, Cuba; 3 Department of Medicine, Faculty of Medicine and Dentistry, University of Alberta, Edmonton, AB T6G 2H7 Canada; 4 Departments of Pediatrics, Division of Medical Genetics and Pediatrics, University of Utah, Salt Lake City, UT 84108 USA

## Abstract

Non-genetic MMP-2 insufficiency is a relatively unexplored condition which could be induced by pathological overexpression of endogenous MMP-2 inhibitors such as TIMPs and/or the acute phase reactant alpha-2-macroglobulin. Here, we investigate the hypothesis that human fibrinogen (FBG) – an acute phase reactant – inhibits human MMP-2. Following an unexpected observation where sera from human donors including arthritis patients with increased levels of serum FBG exhibited reduced binding of serum proMMP-2 to gelatin, we found that human FBG (0 to 3.6 mg/mL i.e., 0 to 10.6 μM) concentration-dependently inhibited human proMMP-2 and MMP2 from binding to gelatin. Moreover, at normal physiological concentrations, FBG (5.29–11.8 μM) concentration-dependently inhibited (40–70% inhibition) the cleavage of fluorescein-conjugated gelatin by MMP-2, but not MMP-9. Indicative of a mixed-type (combination of competitive and non-competitive) inhibition mechanism, FBG reduced the V_max_ (24.9 ± 0.7 min^−1^ to 17.7 ± 0.9 min^−1^, P < 0.05) and increased the Michaelis-Menten constant K_M_ (204 ± 6 n_M_ to 478 ± 50 nM, P < 0.05) for the reaction of MMP-2 cleavage of fluorescein-conjugated gelatin. *In silico* analyses and studies of FBG neutralization with anti-FBG antibodies implicated the domains D and E of FBG in the inhibition of MMP-2. In conclusion, FBG is a natural selective MMP-2 inhibitor, whose pathological elevation could lead to MMP-2 insufficiency in humans.

## Introduction

Matrix metalloproteinase 2 (MMP-2), also known as gelatinase A or 72 kDa type IV collagenase, is a member of a family of 25 different Zn^2+^-dependent endopeptidases involved in the degradation of extracellular matrix proteins (such as collagens)^1,2^ as well as cytokines (such as monocyte chemoattractant protein-3)^3–5^. MMP-2 consists of a pro-peptide domain, a catalytic domain, three fibronectin-like repeats (collagen binding domain) inserted into the catalytic domain and a hemopexin-like (PEX) domain linked to the catalytic domain via a hinge region^6,7^. Proteolytic activity of MMP-2 is regulated at the levels of *MMP2* gene transcription (mRNA synthesis) and translation (protein synthesis), by post translational modifications (such as cleavage of the pro-peptide domain by MT1-MMP) as well as by endogenous inhibitors^1^. These regulatory mechanisms are imperative since both excessive and defective MMP-2 activity can be pro-inflammatory and are implicated in cardiovascular diseases and comorbidities^2,8–12^.

Increased MMP-2 protein expression levels have been associated with increased risk and all-cause mortality in patients with hypertension, ischemic heart disease, heart failure and comorbidities thereof including diabetic and arthritic conditions (particularly rheumatoid arthritis)^8–10,13^. Unfortunately, most previous studies have not specifically correlated MMP-2 expression with MMP-2 activity as determined by MMP-2 binding to or cleavage of important physiological substrates (such as collagens or cytokines). Therefore, it remains unknown whether MMP-2 activity is elevated or not in these conditions despite the reported increase in MMP-2 protein levels, due to a simultaneous increase in levels of endogenous MMP-2 inhibitors. Also, autosomal recessive inactivating mutations of the *MMP2* gene (MMP-2 deficiency) predispose to congenital heart defects, such as transposition of the great arteries to bicuspid aortic valve and septal defects in the atria and ventricles^11^ as well as to a multi-centric osteolysis and arthritis syndrome (MONA; OMIM #259600)^12,14^ – characterized by progressive bone demineralization, destruction of cartilage in joints and abnormal long bone and craniofacial development^15^. Among the endogenous inhibitors of MMP-2 are tissue inhibitors of metalloproteinases (TIMPs)^16,17^ and circulating factors, such as alpha-2-macroglobulin (a positive acute-phase reactant and an anti-protease in the circulation with a broad-spectrum specificity for proteases including MMP-2)^18–22^. Pathological overexpression of these inhibitors could reduce MMP-2 proteolytic activity below normal physiological levels, thus causing MMP-2 insufficiency as observed in liver cirrhosis and pancreatitis patients with high TIMP-2 levels^23^. Similar to alpha-2-macroglobulin, fibrinogen (FBG) is another positive acute-phase reactant in the circulation, where its concentration can increase by up to 10 folds in response to inflammatory stimuli^24^. FBG is a 340 kDa dimeric glycoprotein comprised of two sets of three polypeptide chains (Aα, Bβ and γ) that are interconnected by 29 disulfide bridges^25^. It is unknown whether human FBG inhibits or not human MMP-2 proteolytic activity.

In the present work, we report that high serum FBG levels exhibit impaired binding of serum proMMP-2 to gelatin and selectively inhibits human MMP-2 (but not MMP-9) proteolytic activity by a mixed-type mechanism (i.e., a combination of non-competitive and competitive modes of inhibition). With the aid of *in silico* molecular docking analyses, we found that FBG target domains of MMP-2 involved in catalysis and binding of MMP-2 substrates (collagen peptides). We conclude that FBG is a natural selective MMP-2 inhibitor, whose pathological elevation could contribute to a state of non-genetic MMP-2 activity insufficiency with as-yet poorly understood pathophysiology.

## Results

### Impaired binding of serum proMMP-2 and recombinant MMP-2 to gelatin in the presence of high FBG concentrations

**Figure 1:**
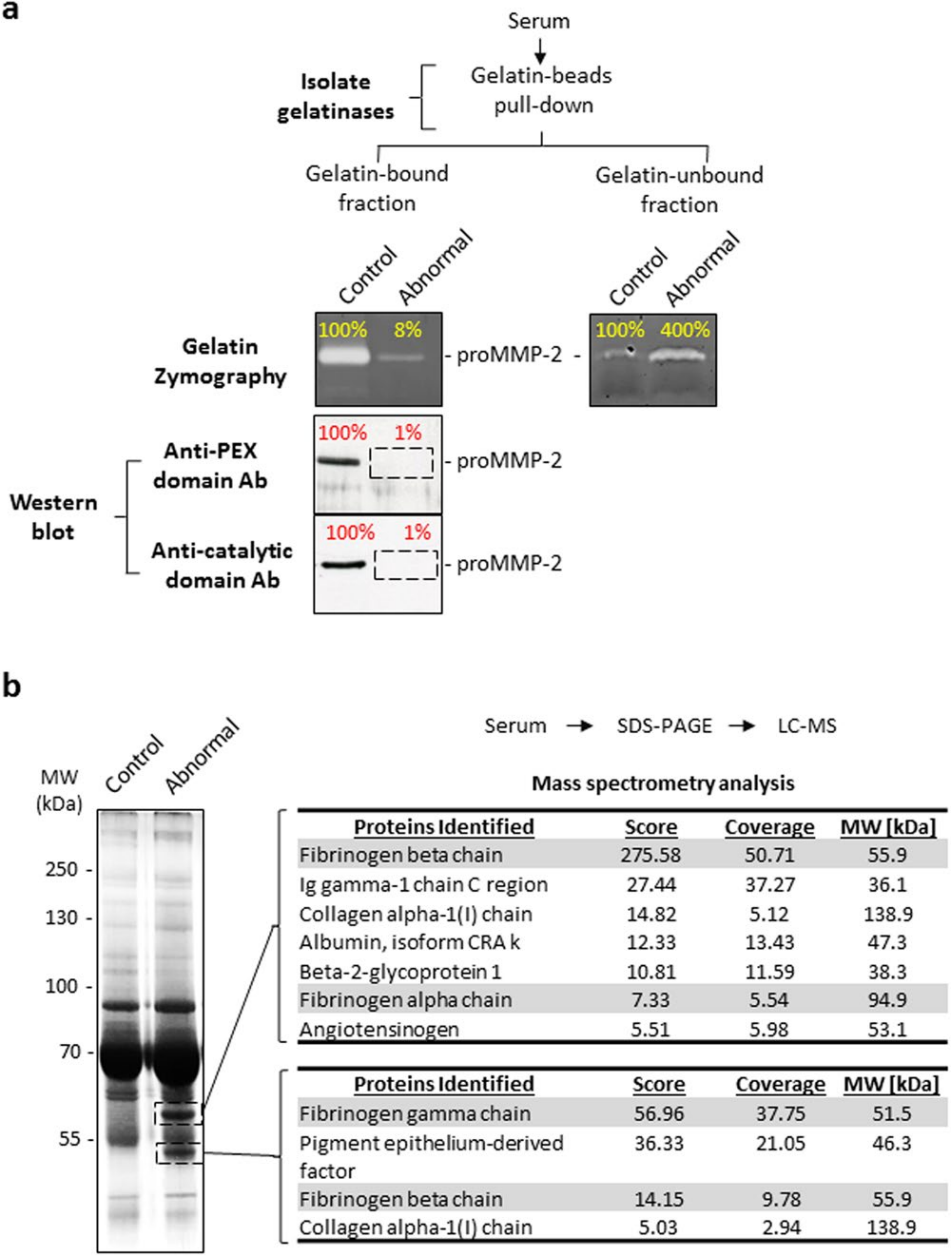
Identification of a blood donor with elevated serum fibrinogen exhibiting impaired binding of MMP-2 to gelatin. (**a**) Analysis of serum samples by gelatin zymography and Western blot to detect MMP-2 and/or to quantitate its gelatinolytic activity. Undiluted serum samples (10 μL) were incubated with an estimated 43 μg of immobilized gelatin at 4 °C for 1 hour. Gelatin-bound and gelatin-unbound proteins were subjected to gelatin zymography to quantitate MMP-2 activity. Gelatin-bound proteins were subjected to Western blot to detect MMP-2 using anti-PEX and anti-catalytic domain (MMP-2) antibodies. Top: Gelatin zymogram showing the majority of activity of serum MMP-2 of the abnormal sample remained in the gelatin-unbound fraction. Bottom: Western blots showing serum MMP-2 of the abnormal sample was undetected in the gelatin-bound fraction by both the anti-PEX domain and anti-catalytic domain antibodies. Band intensities are shown as a percentage relative to the band of the representative control serum sample. Uncropped gels and blots are presented in Supplementary Fig. S7. (**b**) SDS-PAGE coupled with LC-MS (ESI) was used to identify the compositions of two unique protein bands in the abnormal sample as indicated. Uncropped gel is presented in Supplementary Fig. S7. LC-MS, liquid chromatography – mass spectrometry; ESI, electrospray ionization; PEX, hemopexin-like; Ab, antibody; MMP-2, matrix metalloproteinase 2.

While studying human MMP-2 deficiency involving a cohort of MMP-2 deficient patients, rheumatoid arthritis patients and healthy controls (Table S1), we made a serendipitous observation where we identified a control blood donor (denoted herein as abnormal) whose serum proMMP-2 demonstrated an unusual lack of binding to gelatin. We first subjected the serum samples (10 µL undiluted) to a protein separation step using gelatin cross-linked agarose beads (containing 43 µg of immobilized gelatin) to isolate the collagen-binding proteins including proMMP-2. Subsequent gelatin zymography analysis showed that proMMP-2 bound to the gelatin cross-linked agarose beads in all of the tested sera samples with the exception of one serum sample (referred to as abnormal) (Fig. 1a, left). Most of the proMMP-2 of the abnormal sample remained in the gelatin-unbound fraction (Fig. 1a, right), indicating a lack of binding of MMP-2 to gelatin. To confirm this result, we conducted Western blot analyses on the gelatin-bound fractions using two anti-MMP-2 antibodies against two different domains of MMP-2 – the PEX domain (epitope at amino acids 475–490) and the catalytic domain (a region around amino acid P117). Western blots (Fig. 1a, left) detected proMMP-2 in the gelatin-bound fractions of all the sera samples with the exception of the abnormal sample, confirming the results of the zymography analyses, namely that proMMP-2 binding to the immobilized gelatin was reduced in the abnormal sample but not in any of the other serum samples tested (a representative sample is shown in Fig. 1 denoted as control). The donor of the abnormal serum indicated that he was asymptomatic at the time of blood collection and was not clinically investigated.

Next, SDS-PAGE analysis of the sera indicated two prominent protein bands unique to the abnormal serum (not prominent in the other samples), one above 55 kDa and the other at 47 kDa (Fig. 1b, left). Mass spectrometry analysis revealed them to be FBG beta chain (Bβ) and FBG gamma chain (γ) with a high degree of confidence (Fig. 1b, right). FBG concentration in the abnormal sample was 67900 ± 212 ng/mL (as determined by ELISA). These data showed that circulating FBG was elevated in the abnormal sample (reported normal physiological concentration of FBG: 1.8–4 mg/mL (5.29–11.8 µM))^26^.

To ascertain whether the impairment in binding of serum MMP-2 to gelatin (in the donor of the abnormal serum) was reversible (e.g., caused due to a transient elevation of MMP-2 interactors such as FBG in the circulation of the donor) rather than permanent (e.g., due to an inactivating *MMP2* gene mutation), we retested the donor approximately three months after the first blood collection and made the following observations: (i) Serum FBG levels in the donor of the abnormal serum significantly decreased from 67900 ± 212 ng/mL to 1409 ± 247 ng/mL. (ii) MMP-2 binding to gelatin was restored (Fig. S1). These results demonstrated that the binding between MMP-2 and gelatin was restored when FBG was no longer elevated in the serum of the same individual. The cause for the transient elevation in circulating FBG in the donor is unknown and was not investigated.

**Figure 2:**
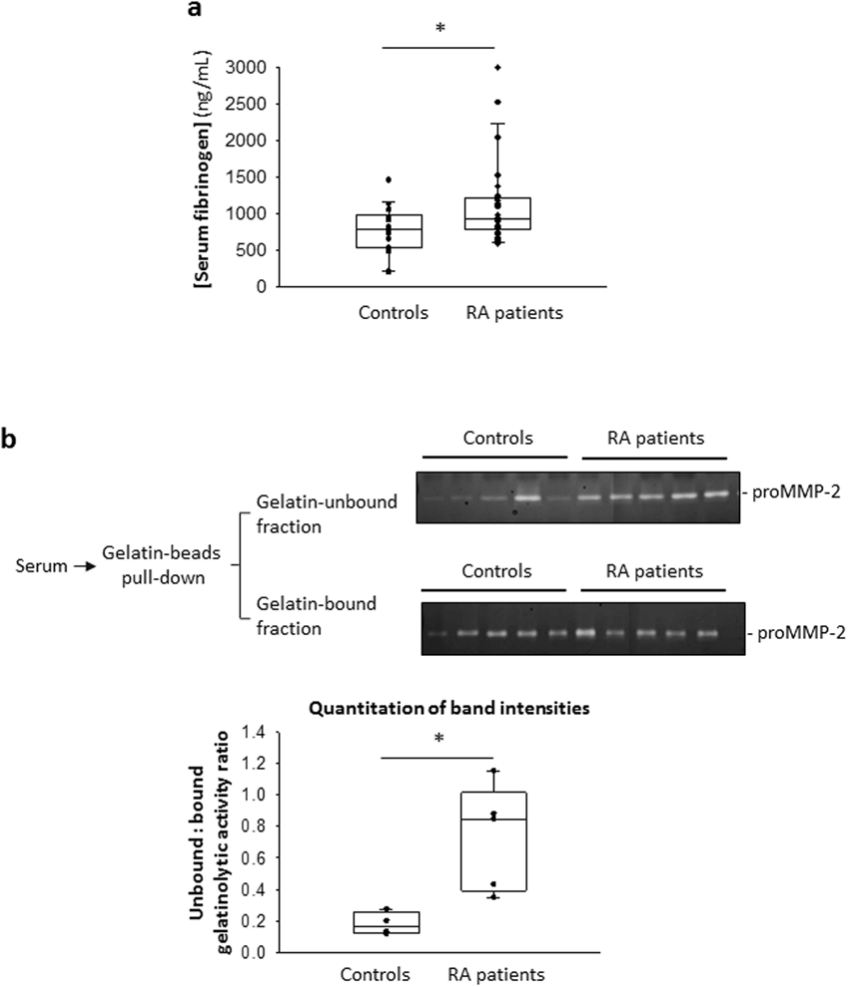
Reduced binding of serum proMMP-2 to gelatin in sera with higher fibrinogen concentrations. (**a**) Quantitation of serum fibrinogen concentrations of rheumatoid arthritis patients (n = 25) and healthy controls (n = 19) by ELISA. P = 0.019 controls vs RA patients, determined by Mann-Whitney Rank Sum Test. (**b**) Top: Gelatin binding assay of selected RA patient (high FBG) and control (low FBG) serum samples, followed by gelatin zymography shows the gelatinolytic activity of proMMP-2 in the gelatin-unbound fraction and the gelatin-bound fraction. Bottom: Quantitation and comparison of the intensities of the lysis bands in the gelatin zymograms above by densitometry. *P < 0.05 controls vs RA patients, determined by Mann-Whitney Rank Sum Test. Uncropped gels are presented in Supplementary Fig. S7. ELISA, enzyme-linked immunosorbent assay; RA, rheumatoid arthritis; FBG, fibrinogen.

**Figure 3:**
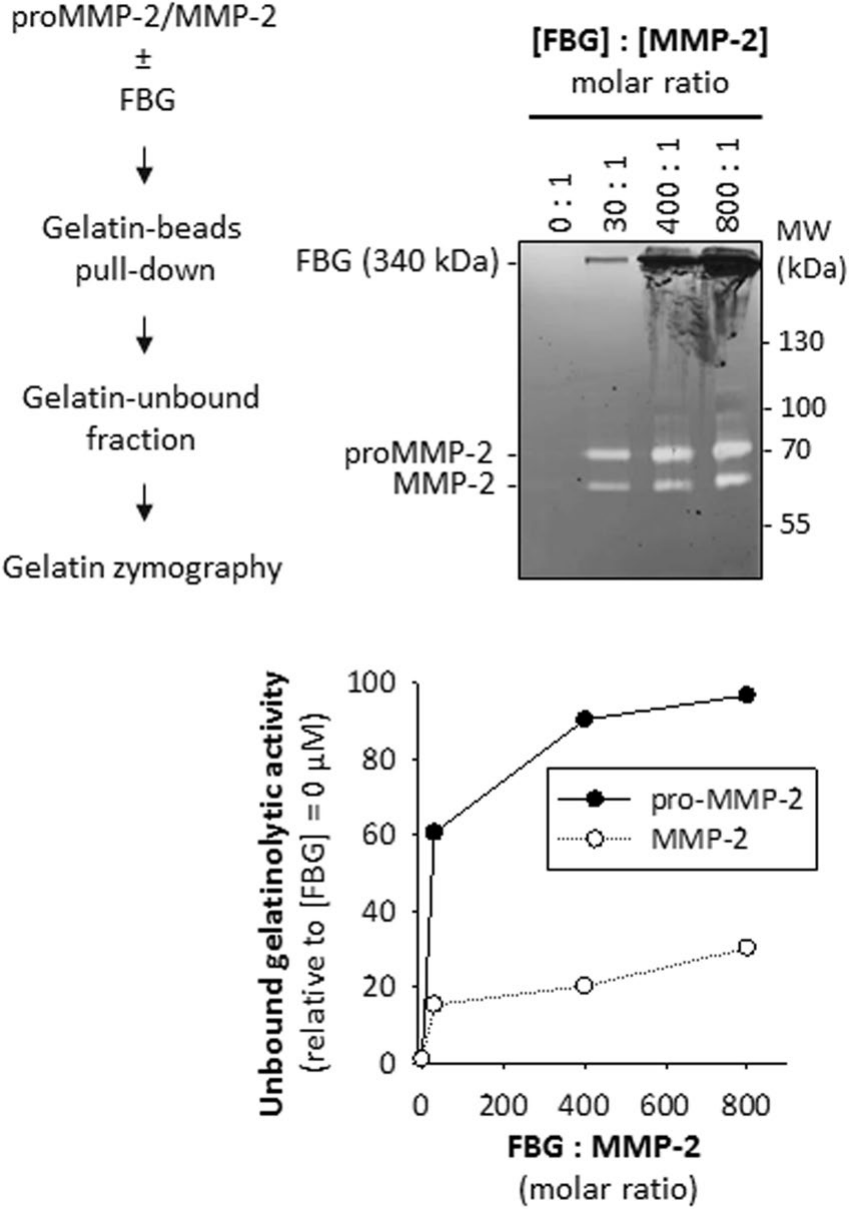
Purified fibrinogen concentration-dependently reduces binding of recombinant MMP-2 to gelatin. Top left: Strategy for assessing a lack of binding of MMP-2 to gelatin in the presence or absence of FBG. Top right: Gelatin binding assay followed by gelatin zymography shows an increase in gelatinolytic activity of MMP-2 remaining in the gelatin-unbound fraction with increasing FBG concentration. Gelatin zymogram showing gelatinolytic activity of MMP-2 in the gelatin-unbound fraction for varying [FBG]: [MMP-2] ratios (0: 1 to 800: 1). Uncropped gel is presented in Supplementary Fig. S7. Bottom: Quantitation of the intensities of the lysis bands in the gelatin zymogram (left) by densitometry. Relative intensity for each band was calculated by dividing the absolute intensity of each band by the absolute intensity of the band at [FBG] = 0 μM ([FBG]: [MMP-2] = 0:1).

Next, we screened sera from rheumatoid arthritis patients and healthy controls to identify samples with elevated serum FBG and performed the gelatin binding assay as described. Rheumatoid arthritis patients exhibiting higher serum FBG levels relative to healthy controls (Fig. 2a) also demonstrated relatively reduced binding of serum MMP-2 to gelatin (Fig. 2b). To test whether FBG indeed disrupted the interaction between MMP-2 and gelatin, we incubated recombinant pro-MMP-2 (0.001 mg/mL; 13.9 nM) with immobilized gelatin (43 µg) in the presence of increasing concentrations of FBG (0 to 3.6 mg/mL; 0 to 10.6 μM) and observed that FBG concentration-dependently inhibited the binding of MMP-2 to its substrate gelatin (Fig. 3).

### Human FBG is not cleaved by recombinant human MMP-2

**Figure 4:**
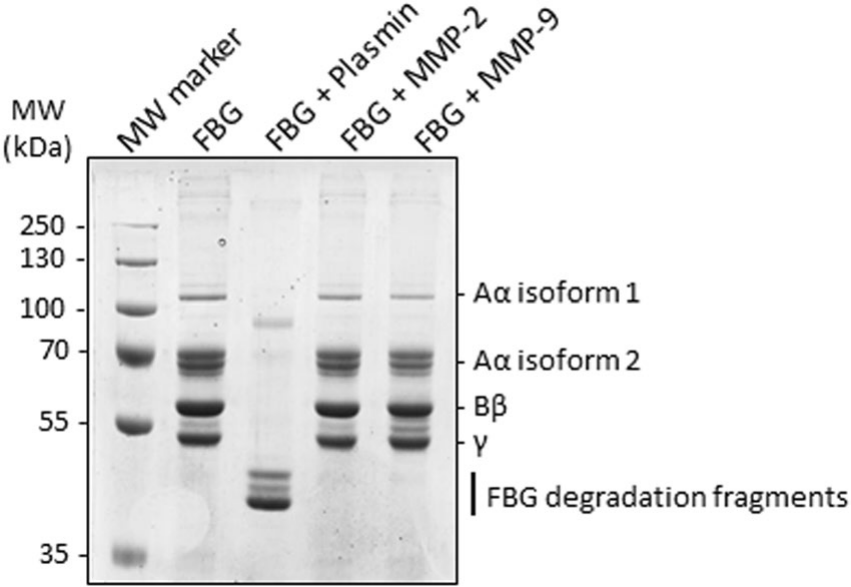
Human fibrinogen is not cleaved by recombinant human MMP-2 or human MMP-9. Proteolysis of human FBG by plasmin, MMP-2 and MMP-9. FBG (3 mg/mL) was incubated with near physiological concentrations of either plasmin (12 nM) or MMP-2 (12 nM) or MMP-9 (12 nM) for 24 hours at 37 °C. The reaction mixtures were analysed by SDS-PAGE.

To test whether any of the three subunits of FBG is cleaved by MMP-2, we incubated purified FBG (3 mg/mL or 8.82 µM) with MMP-2 (12 nM) at 37 °C for 24 hours followed by SDS-PAGE analysis. None of the FBG subunits were cleaved by MMP-2 (nor by MMP-9) whereas all three subunits were degraded by plasmin (12 nM) – a protease that degrades FBG and fibrin in normal physiology^27^ (Fig. 4). As a control to ensure the recombinant MMP-2 used was active when incubated at 37 °C for 24 hours, we included a cleavage reaction with collagen type I as substrate in parallel with FBG (Fig. S2). Although, previous research^28^ reported that the Aα and Bβ chains of bovine FBG are cleaved by MMP-2, the cleavage reported was highly inefficient relative to plasmin mediated degradation of FBG. Our results show that human FBG is not cleaved by recombinant active MMP-2 at physiological concentrations and conditions. This observed lack of cleavage of human FBG by MMP-2 suggests that FBG is not likely to be a physiological target for MMP-2 mediated degradation. Rather, the interaction between FBG and MMP-2 may cause an inhibition of MMP-2 activity – a hypothesis we tested in subsequent experiments.

### FBG inhibits MMP-2 proteolytic activity

**Figure 5:**
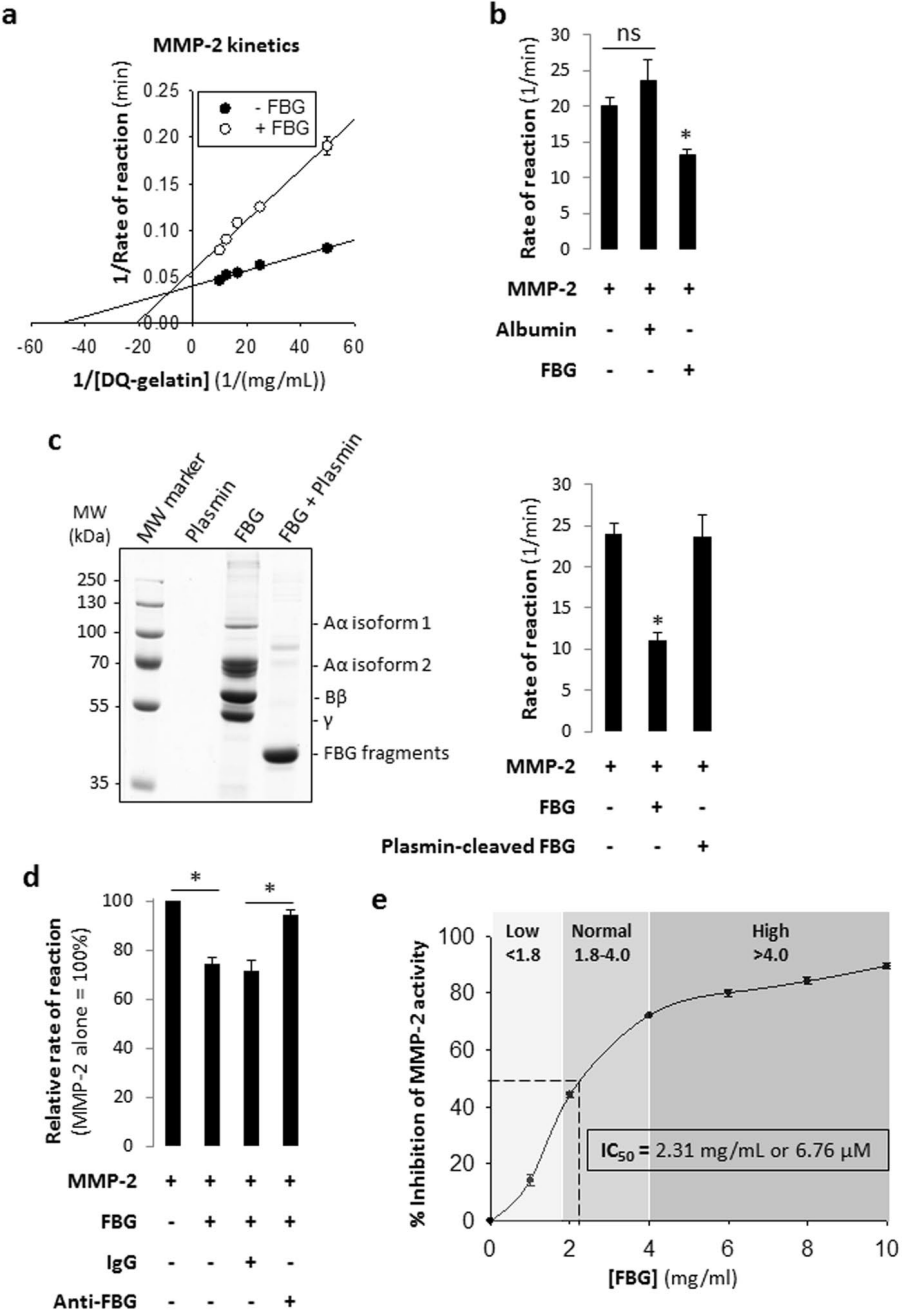
Purified human fibrinogen inhibits gelatinolytic activity of recombinant human MMP-2. (**a**) Lineweaver-Burk plot of the proteolytic processing of DQ-gelatin by MMP-2 in the absence or presence of 3 mg/mL FBG; [MMP-2] = 0.001 mg/mL (13.9 nM) and [DQ-gelatin] = 0.02, 0.04, 0.06, 0.08 or 0.1 mg/mL. (**b**) Bar graphs showing the effect of intact fibrinogen (3 mg/mL) on the activity of MMP-2 (0.001 mg/mL); MMP-2 alone and human serum albumin (3 mg/mL) plus MMP-2 were used as a control. *P < 0.05 vs MMP-2 determined by student’s t-test. ns, not significant. Data are shown as mean of triplicates. (**c**) Left: SDS-PAGE confirming complete degradation of fibrinogen (6 mg/mL) when incubated with plasmin (0.001 mg/mL) for 12 hours at 37 °C. Right: Bar graphs showing that and plasmin-degraded fibrinogen fragments (3 mg/mL) *vs* intact fibrinogen (3 mg/mL) have no effect on the activity of MMP-2 (0.001 mg/mL). *P < 0.05 vs MMP-2 determined by student’s t-test. ns, not significant. (**d**) Bar graph showing restoration of MMP-2 activity when FBG is selectively removed from solution by an anti-FBG antibody. [MMP-2] = 0.001 mg/mL (13.9 nM); [DQ-gelatin] = 0.05 mg/mL; [FBG] = 2 mg/mL (5.88 µM); [IgG] and [anti-FBG] = 5.88 µM. *P < 0.05; determined by two-tailed student’s t-test (n = 4). (**e**) Plot showing the effect of increasing fibrinogen concentrations (0.0 to 10 mg/mL) on MMP-2 (0.001 mg/mL) activity. Note that high circulating FBG concentrations (as those found in RA patients (Fig. 2a)) effectively inhibit MMP-2 activity by more than 50%. Data are presented as mean ± standard error of mean. FBG, fibrinogen; RFU, relative fluorescence unit.

To determine whether FBG inhibits MMP-2 activity, we incubated recombinant active MMP-2 with increasing concentrations of a fluorogenic substrate (fluorescein-conjugated gelatin) in the absence or presence of FBG. Concentrations of MMP-2 and FBG used in the assay –0.001 mg/mL (16.1 nM) and 3 mg/mL (8.82 μM) respectively – were close to their reported normal circulating physiological ranges: 0.00017–0.00049 mg/mL (2.74–8.06 nM) MMP-2^29^ and 1.8–4 mg/mL (5.29–11.8 µM) FBG^26^. The double reciprocal plot (Fig. 5a) of substrate concentration and rate of reaction (change in relative fluorescence unit over time) demonstrates inhibition of MMP-2 activity by FBG. The V_max_ decreased from 24.9 ± 0.7 min^−1^ to 17.7 ± 0.9 min^−1^ (P < 0.05) and the K_M_ increased from 204 ± 6 nM to 478 ± 50 nM (P < 0.05) with the addition of FBG (Table 1), indicating that the mode of inhibition is mixed – a combination of competitive and non-competitive inhibition. As a negative control, substituting human serum albumin (HSA) – the most abundant protein in serum^30^ – in place of FBG did not inhibit MMP-2 activity (Fig. 5b). Next, we demonstrated that after FBG undergoes plasmin-mediated degradation (Fig. 5C, left), the resulting FBG fragments are unable to inhibit MMP-2 and MMP-2 activity was restored (Fig. 5c, right), also suggesting that FBG is required to be intact in order to inhibit MMP-2. Furthermore, neutralization of FBG using an anti-FBG antibody (but not non-immune IgG) lifted inhibition of MMP-2 by FBG, restoring MMP-2 activity (Fig. 5d). A concentration-response experiment (Fig. 5e) showed that FBG concentration-dependently inhibited MMP-2 and the IC_50_ to inhibit the proteolytic activity of 0.001 mg/mL (16.1 nM) MMP-2 was found to be 2.31 ± 0.04 mg/mL (6.76 ± 0.12 μM) FBG. Together, these results clearly demonstrated that FBG inhibits MMP-2 proteolytic activity.

**Table 1:** Summary of kinetic constants of MMP-2 and MMP-9 activity in the absence or presence of fibrinogen.

Kinetic constants	−FBG	+FBG	P-value
**MMP-2**			
K_M_ (nM)	204 ± 6	478 ± 50	0.007
V_max_ (min^−1^)	24.9 ± 0.7	17.7 ± 0.9	0.003
**MMP-9**			
K_M_ (nM)	272 ± 7	240 ± 10	0.118
V_max_ (min^−1^)	111 ± 4	98 ± 3	0.0815

**Figure 6:**
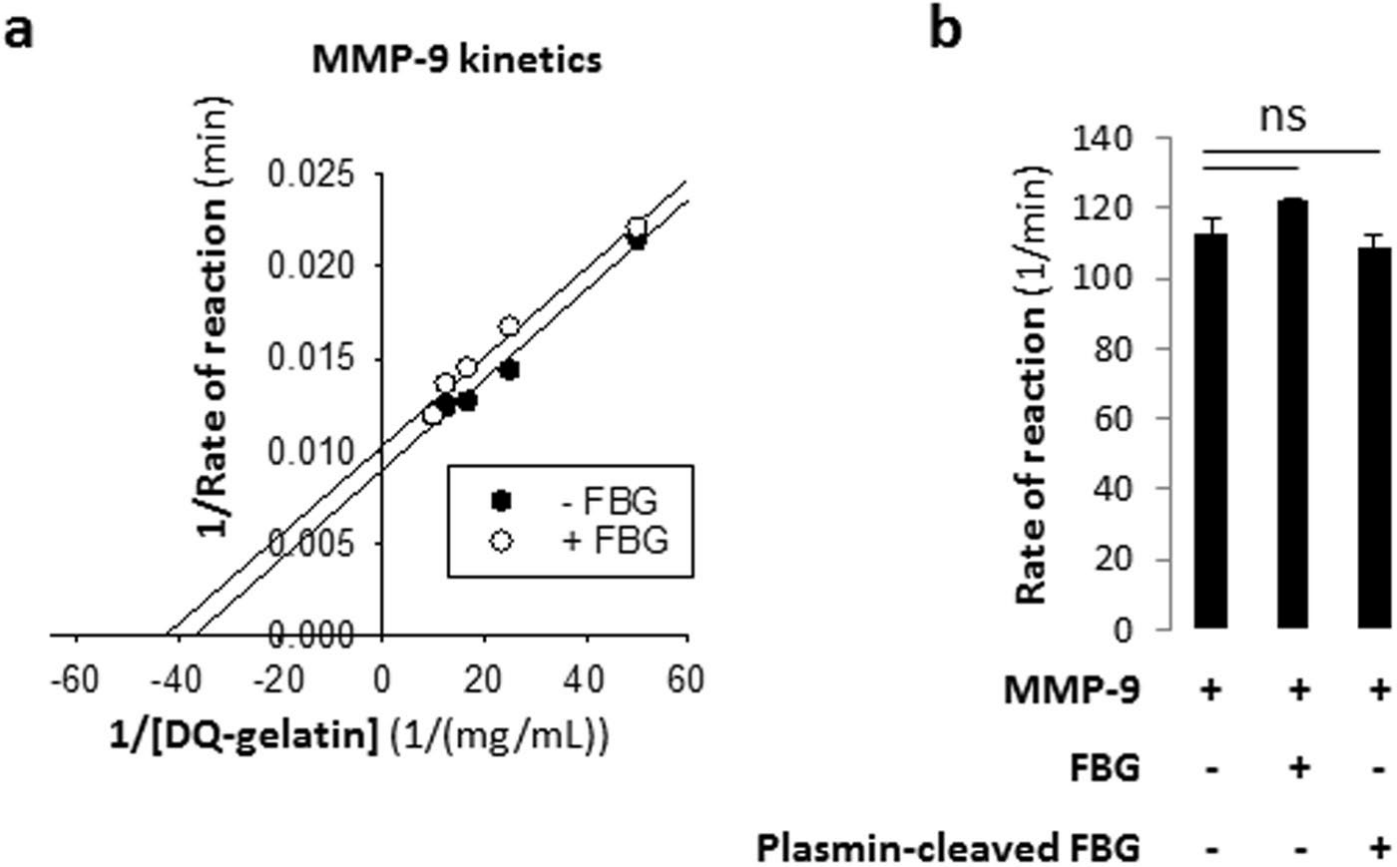
Gelatinolytic activity of recombinant human MMP-9 is not inhibited by purified human fibrinogen. (**a**) Lineweaver-Burk plot of the proteolytic processing of DQ-gelatin by MMP-9 in the absence or presence of 3 mg/mL FBG; [MMP-9] = 0.001 mg/mL (12.0 nM) and [DQ-gelatin] = 0.02, 0.04, 0.06, 0.08 or 0.1 mg/mL. (**b**) Bar graphs showing that intact fibrinogen (3 mg/mL) or plasmin-degraded fibrinogen fragments (3 mg/mL) has no effect on the activity of MMP-9 (0.001 mg/mL). ns, not significant. Data are presented as mean ± standard error of mean. FBG, fibrinogen; RFU, relative fluorescence unit.

Maximum velocity (V_max_) and the Michaelis-Menten constant (K_M_) derived from the Lineweaver-burk plots presented in Figs 5a and 6a. Data are presented as mean ± standard error of mean (n = 3). Comparison of +FBG vs −FBG, P-value determined by two-tailed student’s t-test.

To test whether the inhibitory actions of FBG may also be applicable to other MMPs, we tested using the same flourometric gelatinase activity assay the effects of FBG on MMP-9 – another gelatinase of the MMP family that is structurally homologous to MMP-2^31^. The double reciprocal plot (Fig. 6a) of substrate concentration and rate of reaction showed no significant change in either the V_max_ (111 ± 4 min^−1^ *versus* 98 ± 3 min^−1^; P > 0.05) or the K_M_ (272 ± 7 nM *versus* 240 ± 10 nM; P > 0.05) with the addition of FBG (Table 1), demonstrating that intact FBG does not inhibit MMP-9 activity. Also, MMP-9 proteolytic activity was not inhibited by plasmin-cleaved FBG (Fig. 6b).

### FBG targets the catalytic domain of MMP-2

**Figure 7:**
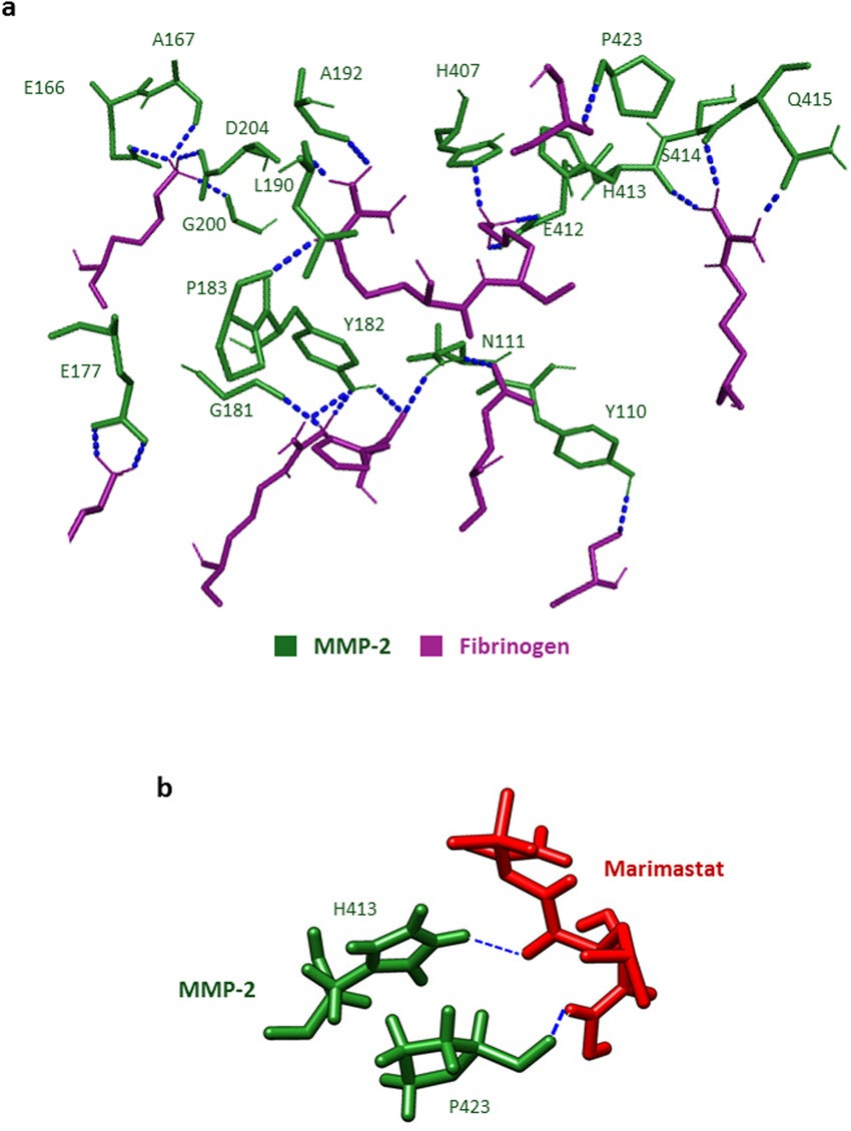
Fibrinogen and Marimastat bind at a common region of the catalytic domain of MMP-2. (**a**) Molecular docking of MMP-2 to FBG. Labelled residues of MMP-2 (green) that form hydrogen bonds (blue dotted lines) with FBG residues (magenta) are presented. (**b**) Molecular docking of MMP-2 to Marimastat. Labelled residues of MMP-2 (green) that form hydrogen bonds (blue dotted lines) with Marimastat (red) are presented.

To understand the molecular mechanism of interaction between FBG and MMP-2 which results in the inhibition of MMP-2 activity, we performed *in silico* molecular docking analyses which revealed that FBG binds MMP-2 at the catalytic domain. Docking of FBG to MMP-2 showed that the interaction between FBG and MMP-2 is stabilized via hydrogen bonds and non-polar interactions between residues in the catalytic domain of MMP-2 and the D domain of FBG (Figs 7a and S3, Table S2). Docking of Marimastat – a synthetic MMP-2 inhibitor that targets the catalytic domain – to MMP-2 revealed two MMP-2 residues (His413 and Pro423) that form hydrogen bonds with Marimastat (Fig. 7b, Table S2). These two MMP-2 residues are also involved in stabilizing FBG-MMP-2 interactions via hydrogen bonds, thus showing that FBG and Marimastat bind at a common region of the catalytic domain of MMP-2. Moreover, comparing docking models of the FBG-MMP-2 complex and the collagen peptide-MMP-2 complex (Fig. S4, Table S3) revealed that FBG and collagen interact with several common MMP-2 residues at the catalytic domain, namely R127, G165, E166, M170, D180, G200, V201 and D204. Together, these results predict FBG to interact with MMP-2 at regions in the catalytic domain where the synthetic MMP-2 inhibitor Marimastat and the MMP-2 substrate collagen (peptide) bind.

## Discussion

We began this investigation when we made the serendipitous observation that a blood donor with abnormally high concentration of serum FBG displayed reduced binding of serum proMMP-2 to gelatin-coated beads. To clarify whether pathological elevation of FBG leads to reduced binding of serum proMMP-2 to gelatin, we further tested sera from rheumatoid arthritis patients – a pathology associated with high serum FBG levels. Interestingly, binding of proMMP-2 to gelatin-coated beads was significantly reduced in the rheumatoid arthritis patients as well. Based on these observations, we hypothesized that FBG is an inhibitor of MMP-2. Through *in vitro* studies, we confirmed reduced binding of recombinant human proMMP-2 (72 kDa) as well as active MMP-2 (62 kDa) to gelatin-coated beads in the presence of purified human FBG. Subsequent Lineweaver-burk enzyme kinetics analyses demonstrated that human FBG is a natural mixed-type (i.e., competitive and non-competitive) selective inhibitor of MMP-2 (but not MMP-9). *In silico* analyses and studies of FBG neutralization with anti-FBG antibodies implicated the domains D and E of FBG in the inhibition of MMP-2. Our data indicate that FBG is a natural MMP-2 inhibitor and a pathological elevation of circulating FBG could limit MMP-2 binding to and cleavage of important physiological substrates.

Elevated FBG expression indicates inflammation in infections, arthritis, atherosclerosis, heart failure, and kidney failure, as well as being mechanistically implicated in thrombosis^24,32,33^. FBG and its derivative peptides also function as chemoattractants for leukocytes^32,34^. MMPs, including MMP-2, are well known modulators of immune cell chemoattraction^1,2,35^. However, the interplay between FBG and MMPs in the settings of the above inflammatory conditions remains poorly understood. MMP-2 deficiency causes inflammation amid skeletal and cardiac disorders^2,12,15^, suggesting that prolonged inhibition of MMP-2 activity by FBG, and perhaps also by other acute phase reactants (as is the case of alpha-2 macroglobulin^18^) or TIMPs, may be pro-inflammatory as well. However, it cannot be excluded that the elevation of FBG plays anti-inflammatory role in pathologies for which MMP-2 expression leads to increased disease activity. Interestingly, we have found that normal physiological concentrations of circulating FBG are sufficient to inhibit circulating concentrations of MMP-2 (by 40–70%). Conceivably, the inhibition of circulating MMP-2 activity by FBG serves to downregulate platelet activation and aggregation. Indeed at sites of vascular injury, platelets release MMP-2 which, in turn, potentiates platelet activation by interacting with integrin α_IIb_β_3_ on the platelet surface via its PEX domain^36–38^. Active MMP-2 is also able to facilitate platelet aggregation triggered by agonists such as adenosine diphosphate (ADP), collagen or thrombin^39^. FBG displaces MMP-2 and binds to the ectodomains of integrin α_IIb_β_3_ molecules on the surface of platelets to tether platelets together, thus stabilizing platelet aggregates^37,40^. The binding of free FBG molecules to proteolytically active MMP-2, resulting in inhibition of MMP-2 activity, could lead to downregulation of the potentiation of platelet activation via MMP-2 activity dependent mechanisms. We have demonstrated that FBG has no effect on MMP-9 activity, which might favour the downregulation of platelet activation as MMP-9 is able to counteract the pro-aggregatory effects of MMP-2^36,41,42^. In line with previous research^37,40^, FBG, while stabilizing platelet aggregates, might also play an anti-aggregatory role in MMP-2 mediated platelet activation by inhibiting MMP-2 activity – a hypothesis worth investigating in future studies (Fig. S5).

Besides the inhibition of circulating MMP-2, FBG could also inhibit interstitial MMP-2 activity in conditions where FBG is deposited in the extracellular matrix without subsequent conversion to fibrin, such as at sites of tumorigenesis^43–45^. Interstitial MMP-2 activity downregulates fibrosis – excess deposition of a collagen-rich extracellular matrix that can occur in most organs^46^. Moreover, accumulation of interstitial FBG upregulates fibrosis by acting as a pro-fibrotic ligand^47^, by increasing TGF-β1 production in resident macrophages in skeletal muscles^48^ as well as by binding to ICAM-1 in target cells^49^. Conceivably, a possible mechanism by which FBG upregulates fibrosis could be via inhibition of anti-fibrotic action of MMP-2 – a hypothesis that warrants further investigation.

How selective is human FBG as an inhibitor for human MMP-2? To address this question, we tested human MMP-9 – the closest member of the MMP protein family to MMP-2 in terms of structural homology as well as sequence identities of their catalytic domain, fibronectin type II repeats and the PEX domain^31^. We found that human MMP-9 activity was not inhibited by human FBG. Sequence alignments of the catalytic domain and the PEX domain of different members of the MMP family showed low similarity between MMP-2 and other MMPs (Fig. S6). Based on the sequence alignments, we predict that human FBG selectively inhibits human MMP-2 and does not inhibit other human MMPs, as we have confirmed for human MMP-9. We do not know if FBG from other species will inhibit human MMP-2, as human FBG does.

Of note, we are aware that Monaco, S. *et al*.^28^ investigated the hydrolysis of bovine FBG by human MMP-2. However, our study has focused on the interaction between human FBG and human MMP-2. To explain a potential inter-species difference in the ability of MMP-2 to cleave FBG of bovine but not of human origin, we examined the alignment of amino acid sequences of the subunits of bovine *versus* human FBG. The analyses show that the sequence similarities of Alpha, Beta and the Gamma subunits are at 58.2%, 82.4% and 83%, respectively. We think that the differences in the amino acid sequences between human and bovine FBG are likely responsible for the different sensitivities to MMP-2 cleavage exhibited by FBG from these species.

Having investigated the selectivity of FBG for MMP-2, we explored the interaction mechanism between FBG and MMP-2 using *in silico* molecular docking analyses and domain-specific anti-FBG antibodies. Our *in silico* analyses showed that FBG binds to MMP-2 at the catalytic domain and interacts with the same MMP-2 residues that interact with the synthetic MMP-2 inhibitor – Marimastat. Specifically, both FBG and Marimastat interact with the MMP-2 residues F184, G184, A192, H193, H407, H413, P423, I424 and Y425 via either polar or non-polar bonds. Moreover, FBG and collagen interact with several common MMP-2 residues, namely R127, G165, E166, M170, D180, G200, V201 and D204, indicating that FBG, when bound to MMP-2, could block collagen (and perhaps other MMP-2 substrates) from accessing the active site in the catalytic domain of MMP-2. The interaction of FBG with MMP-2 spans over the active site in the catalytic domain as well as the fibronectin type II repeat 2, supporting the conclusion we derived from our experimental data that the mode of MMP-2 inhibition by FBG is mixed-type (FBG competes with MMP-2 substrates for the catalytic domain as well as binds to residues in the fibronectin type II repeat 2 domain). A plausible mechanism of the non-competitive inhibition is that FBG binds to MMP-2 or MMP-2-substrate complex at the fibronectin type II repeat 2 (or at a different site not predicted by the *in silico* analyses) and prevents catalysis by altering the molecular conformation of MMP-2. Further, our data indicate that adding anti-FBG antibody but not IgG enables MMP-2 to exert gelatinase activity in the presence of FBG. Anti-FBG binds to domains D and E of FBG (as per the manufacturer of this antibody). The *in silico* data gathered in this investigation predicts domain D to be involved in the interactions between FBG and MMP-2. Together, these data further support the notion that FBG inhibits MMP-2 via domains D or E. Consistently, complexation of FBG with anti-FBG inhibits FBG-mediated inhibition of MMP-2, whereas non-immune IgG (control) has no such effect.

In conclusion, this investigation identifies human FBG as a selective mixed-type inhibitor of human MMP-2. The pathological elevation of circulating FBG, together with other known MMP-2 inhibitors, could lead to a state of non-genetic MMP-2 insufficiency which may cause pathologies including arthritic and cardiac disorders reminiscent of those described for patients with genetic MMP-2 deficiency^12,15^. We believe that this study is a starting point in a new direction of research on the possible occurrence of conditions caused by MMP-2 activity insufficiency amid apparently normal levels of MMP-2 expression – the potential clinical implications of such conditions are as yet unknown and merit further research.

## Materials and Methods

The serum samples used in this study were collected at the University of Utah Hospital (Salt Lake City, UT) and the University of Alberta Hospital (Edmonton, AB) with informed consent from the donors. The study was conducted with approval from the Health Research Ethics Board (HREB) at the University of Alberta. Healthy controls consisted of 17 females and 5 males whereas RA patients consisted of 20 females and 5 males, between the ages of 34 and 77 years (Table S1). The abnormal serum sample belonged to an asymptomatic 30 year old male. Collagen derivatives (gelatin and fluorescein-conjugated gelatin) were used as surrogate for collagen in *in vitro* assays. All (following) methods were performed in accordance with the relevant guidelines and regulations.

### Serum MMP-2 isolation using immobilized gelatin

Serum MMP-2 was isolated on gelatin immobilized on cross-linked 4% agarose beads (Sigma, Missouri, USA, cat# G5384). The beads suspension was spun down at 2000 × g for 2 mins in a bench-top centrifuge (Mandel mini, Mandel Scientific, CA) and the supernatant was removed. 40 µL of packed gelatin-beads were washed three times with 1 mL of ice-cold phosphate-buffered saline (PBS; pH 7.4, Thermo Fisher Scientific, Massachusetts, USA) and resuspended in 120 µL of PBS. 10 µL of each serum sample was added to 40 µL of the beads suspension (1: 4, v:v) in separate 0.5 mL microcentrifuge tubes and incubated at 4 °C for 1.5 hours with shaking at 1300 rpm in a Thermomixer R (Eppendorf^TM^). After centrifugation (at 2000 × g for 2 mins), the supernatant (containing proteins not bound to gelatin-beads) was separated (using a pipette) from the beads (containing gelatin-bound proteins including MMP-2) and collected in new microcentrifuge tubes. The beads remaining in the tubes were washed 3 times with 1 mL of PBS, resuspended in 50 µL of PBS and stored on ice until subsequent zymography and Western blot analyses.

### Quantitation of serum FBG concentration

We used an enzyme-linked immunosorbent assay (ELISA) kit (cat# ab208036, Abcam) to quantitate the concentrations of serum FBG in the donors, following manufacturer’s instructions provided with the kit.

### MMP-2 quantitation by substrate zymography analyses

Gelatin zymography system: porcine skin gelatin (Sigma, cat# G8150) was copolymerized with the 10% SDS-PAGE gel (at a final gelatin concentration of 0.2% v:v). Equal volumes of a non-reducing sample buffer (62.6 mM Tris-HCl, pH 7.4, 25% (v/v) Glycerol, 4% (w/v) SDS and 0.01% bromophenol blue) were added to the samples (unfractionated serum, recombinant MMP-2 or gelatin- bound or unbound fraction). Gels were run using vertical gel electrophoresis apparatus (Amersham Biosciences), at 200 V constant for 2 hours. The gel was washed three times with 2.5% (v/v) Triton X-100 for 20 mins each time and incubated at 37 °C overnight (12 h) in an enzyme assay buffer (25 mM Tris-HCl pH 7.4, 5 mM CaCl_2_, 150 mM NaCl, 0.05% (v/v) Brij^TM^-35). The gels were stained with Coomassie blue and destained in 25% (v/v) methanol/10% (v/v) acetic acid. MMP-2 activity towards either gelatin or fibrinogen resulted in clear substrate-lysis bands contrasting against a blue background (from Coomassie staining) in the gel. Intensities of the lysis bands were determined by densitometric scanning using the software ImageJ (NIH, Bethesda, MD).

### Protein binding assay to assess effect of FBG on gelatin - MMP-2 interaction

Lyophilised human FBG (Sigma, cat# F3879) was reconstituted in PBS at 10 mg/mL concentration (estimated [FBG] = 6 mg/mL or 17.6 µM) to make a working solution. 40 µL of packed gelatin-beads were washed three times with 1 mL of PBS and resuspended in 40 µL of PBS. Increasing concentrations of FBG (0, 1.2, 1.8, 3.6 mg/mL) were pre-incubated with a constant 0.001 mg/mL recombinant proMMP-2 (Calbiochem) at 4 °C for 30 mins in 0.5 mL centrifuge tubes with shaking (1100 rpm) in a thermomixer R (Eppendorf^TM^). Equal volumes of gelatin-beads (containing an estimated 43 µg gelatin in 10 µL) were then added and the mixture was incubated for a further 1.5 hours in the same conditions. The mixture was then centrifuged (2000 × g, 1–2 mins) - until all the beads had settled at the bottom of the centrifuge tubes. The supernatant (the gelatin-unbound fraction) was separated and analyzed for gelatinase activity using the substrate zymography method described above.

### Western immunoblotting to detect MMP-2

Serum and recombinant MMP-2 was detected by Western blot. Recombinant proMMP-2 (Calbiochem) was included as a positive control for detection by primary anti-MMP-2 antibodies. Equal volumes of samples were mixed with a reducing sample buffer (150 mM Tris-HCL, pH 6.8, 15% (w/v) SDS, 30% (v/v) glycerol and 10% (v/v) 2-mercaptoethanol), heated at 95 °C for 10 mins and run on a SDS/10%-PAGE. The gel was stained with the Zn-Imidazole reverse stain technique previously described by us^50,51^. After imaging the gel, the Zn-Imidazole stain was removed from the gel by Zn chelation; i.e., by incubating the gel in 50 mM EDTA/ 1X running buffer (25 mM Tris, 192 mM glycine and 3.4 mM SDS). For Western immunoblotting, the proteins were transferred from the gel onto a 0.2 µm nitrocellulose membrane (BioRad, USA). The membrane was then probed with a rabbit polyclonal anti-MMP-2 antibody (Abcam, cat# ab37150) or a rabbit monoclonal anti-MMP-2 (D2O4T) antibody (CellSignalling, cat# 87809). These primary antibodies were then detected using a horseradish-peroxidase-conjugated goat anti-rabbit secondary antibody (BioRad, USA) and the Amersham ECL Western Blot Detection Reagent (GE Healthcare, cat# RPN2106).

### Serum protein identification by LC–MS

Identification of proteins in serum was done by liquid chromatography/mass spectrometry starting with SDS-PAGE-resolved Zn-Imidazole stained protein bands. The gel was excised and de-stained in 50 mM EDTA (pH 8) for 10 mins and washed in deionized water. The gel pieces were washed with 100 mM ammonium bicarbonate/acetonitrile (v/v, 50:50), reduced (with 10 mM 2-mercaptoethanol in 100 mM bicarbonate) and alkylated (with 55 mM iodoacetamide in 100 mM bicarbonate). Gel pieces were dehydrated using 100% acetonitrile and protein in the gel was digested with trypsin (6 ng/μL) for 16 hours at 25 °C. Eluted tryptic peptides were collected. Residual peptides in the gel pieces were recovered by two consecutive extractions in: i) extraction buffer A (97% water/2% acetonitrile/1% formic acid) and ii) extraction buffer A supplemented with acetonitrile (1:1, v/v). Peptide extracts were combined and subjected to protein identification. For protein identification, the combined peptides were analysed by liquid chromatography (Easy-nLC II, Thermo Scientific) and mass spectrometry (LTQ XL-Orbitrap hybrid mass spectrometer (Thermo Scientific)). Mass data analysis was conducted using the Proteome Discoverer 1.4/SEQUEST platform for proteome analysis (Thermo Scientific), at the Alberta Proteomics and Mass Spectrometry Facility (University of Alberta).

### MMP-2 activity determination by a flourometric enzyme activity assay

The effect of human plasma FBG (Sigma) on the proteolytic activity of recombinant human MMP-2 (Abcam, cat# ab81550) was determined using the Enzcheck® gelatinase/collagenase assay kit (Thermo Fisher Scientific). DQ^TM^ gelatin from pig skin conjugated with fluorescein was used as the MMP-2 substrate whose final concentrations were varied from 0.02 mg/mL to 0.1 mg/mL. The concentrations of MMP-2 and FBG used in the assay were kept constant at 0.001 mg/mL and 3 mg/mL respectively. The final assay volume was 100 µL and contained 50 mM Tris-HCl (pH 7.6), 150 mM NaCl, 5 mM CaCl_2_, 0.2 mM NaN_3_. The assay was performed in SpectraPlate^TM^ -384 MB microplates (PerkinElmer). MMP-2 was pipetted to wells containing increasing concentrations of the gelatin substrate (0.02, 0.04, 0.06, 0.08 and 0.1 mg/mL) either in the presence or absence of FBG. Corresponding negative controls (no MMP-2 added) were set up. The contents of each well were mixed by pipetting the mixture up and down. Fluorescence was measured using the Synergy^TM^ H4 hybrid microplate reader (BioTek), at 37 °C for 5 hours with fluorescence readings taken every 60 seconds. Read mode was set to top optic with gain 50, and the excitation and emission wavelengths were set to 485 nm and 528 nm, respectively. For each time point, the background fluorescence was corrected by subtracting the measured fluorescence value derived from the no-enzyme (negative) control. The corrected fluorescence values of each reaction condition were plotted against time and the rates of reaction were determined by calculating the initial gradient once the curves reached linearity (when the rate of product formation is highest). As a negative control, human serum albumin (HSA; Sigma, cat# A3782) was used instead of FBG.

### Restoration of MMP-2 activity by neutralization of FBG

We tested the effect of neutralizing FBG in solution on MMP-2 activity using two complimentary methods: (i) FBG (6 mg/mL in 1x PBS, pH 7.4) was degraded by incubating with 0.001 mg/mL purified human plasmin (Sigma, cat# P1867) for 12 hours at 37 °C. The degradation of FBG was confirmed by SDS-PAGE and the fragments were subsequently used to assess their effect on MMP-2 activity in the gelatinase activity assay described above. (ii) FBG (4 mg/mL in 1X PBS, pH 7.4) was incubated with equimolar concentrations (11.76 µM) of polyclonal rabbit anti-human fibrinogen (anti-FBG; Agilent Dako) at 37 °C for 2 hours to form an immunocomplex. In a control experiment, we incubated FBG with non-immune negative control rabbit immunoglobulin (IgG; Dako Agilent) which does not complex with FBG. Following brief centrifugation (2000 × g for 2 mins), the FBG solutions (final [FBG] = 2 mg/mL) containing either anti-FBG or IgG were subsequently used to assess their effect on MMP-2 activity in the gelatinase activity assay described above.

### *In silico* molecular modelling and protein-protein docking

Crystal structures of full length proMMP-2 (PDB ID: 1CK7)^52^, dimeric form of human fibrinogen (PDB ID: 3GHG)^53^ and a collagen model peptide (PDB ID: 1BKV)^54^, and Marimastat (PubChem CID: 119031)^55^ were used for *in silico* molecular protein-protein docking analyses. To simulate a catalytically active MMP-2, the propeptide (amino acids 30–109) of full length proMMP-2 was removed using Python Molecular Viewer (MGLTools software, The Scripps Research Institute). The generated structure was then refined using 3Drefine server^56,57^ and used for subsequent docking with FBG, collagen and Marimastat. Docking of MMP-2 with FBG and collagen was done using the online server ClusPro with no manual changes in default settings described by the developers^58–62^. We selected the best model based on the most negative binding energy and the highest cluster size. Marimastat docking to MMP-2 was performed following the procedure previously described by Jha *et al*.^63^ using Chimera UCSF^64^ and AutoDock Vina 1.1.2^65^. The structure of Marimastat was minimized using the default parameters (steepest descent steps: 100; steepest descent step size (Å): 0.02, conjugate gradient steps: 10; conjugate gradient step size (Å): 0.02; update interval: 10; fixed atoms: none) on Chimera, hydrogens were added and Gasteiger charges were assigned for a net charge of +0. MMP -2 structure preparation included solvent deletion, addition of hydrogens and assignment of Gasteiger charges. The AutoDock Vina docking grid map was set to cover the full structure of MMP-2 with the maximum number of binding modes (set at 10) and maximum exhaustiveness of search (set at 8). The best ranked model was selected based on the predicted binding affinity in kcal/mol, RMSD values and hydrogen bonds. Polar contacts in the docked models of collagen with MMP-2, FBG with MMP-2 and FBG with MMP-2 docked to collagen were identified using PyMOL (Schrödinger, LLC, NY). Molecular interactions were determined using the software CONTACT of CCP4^66^ to find potential interacting residues within 5Å. Interactions were classified as hydrogen bonds or other (polar or non-polar) interactions.

### Sequence alignment

The sequences of the PEX and the catalytic domains of MMPs were obtained from UniProt (The UniProt Consortium). Sequences were aligned using the protein alignment tool blastp (NCBI, Bethesda, MD, USA).

### Statistical analysis

The results were analyzed and the graphs were plotted using SigmaPlot 13 (Systat Software, San Jose, CA). Data are presented as mean ± standard error of mean. Student’s t-test analysis or Mann-Whitney Rank Sum Test was conducted, where appropriate (indicated in the figure legends), to determine statistical significance in the difference between two groups.

## Supplementary information

Supplementary Information

## Data Availability

The datasets generated during this study are available from the corresponding author upon request.
