# Supplementary material for: Identification of fibrinogen as a natural inhibitor of MMP-2
Source: Sci Rep. 2019 Mar 13;9:4340. doi: 10.1038/s41598-019-40983-y (PMC6416301; doi:10.1038/s41598-019-40983-y)
Supplement: Supplementary file 1 — Supplementary Information [file 41598_2019_40983_MOESM1_ESM.pdf]

Identification of fibrinogen as a natural inhibitor of MMP-2

Authors:

Hassan Sarker (1), Eugenio Hardy (1, 2), Ayman Haimour (1), Walter P. Maksymowych (3), Lorenzo D. Botto (4), Carlos Fernandez-Patron (1*)

Authors' affiliations:

(1): Department of Biochemistry, Faculty of Medicine and Dentistry, University of Alberta, Edmonton, AB T6G 2H7, Canada.

(2): Biotechnology Laboratory, Study Center for Research and Biological Evaluations, Institute of Pharmacy and Foods, University of Havana, Havana, P.O. Box 13600, Cuba.

(3): Department of Medicine, Faculty of Medicine and Dentistry, University of Alberta, Edmonton, AB T6G 2H7, Canada.

(4): Departments of Pediatrics, Division of Medical Genetics and Pediatrics, University of Utah, Salt Lake City, UT 84108, USA.

(*): Correspondence:

Carlos Fernandez-Patron: cf2@ualberta.ca

Department of Biochemistry

Faculty of Medicine and Dentistry

University of Alberta

Edmonton, AB T6G 2H7, Canada

Running title: Fibrinogen inhibits MMP-2

Key words: Matrix metalloproteinase, MMP-2, Fibrinogen, MMP-2 deficiency

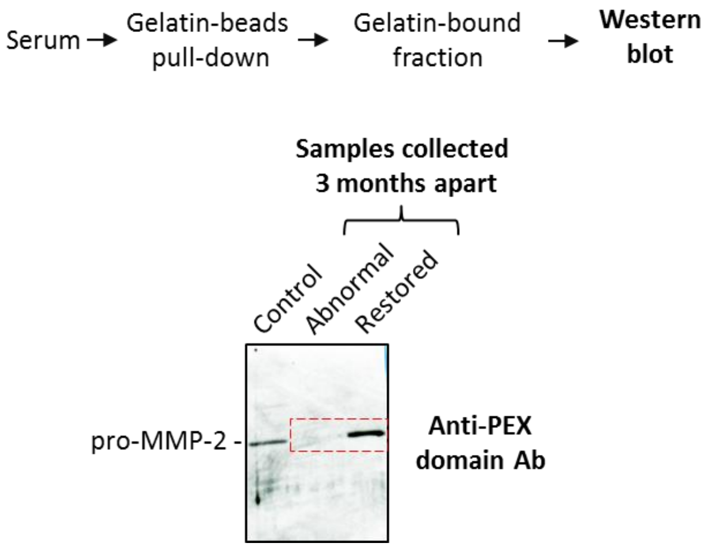

Figure S1: Restoration of binding of serum MMP-2 to gelatin when serum fibrinogen levels normalized. Western blot (using the anti-PEX domain antibody) showing MMP-2 in the gelatin-bound fraction indicating the restoration of binding of MMP-2 to gelatin in a second serum sample from the donor of the abnormal serum collected three months after the first collection. PEX, hemopexin-like domain; Ab, antibody.

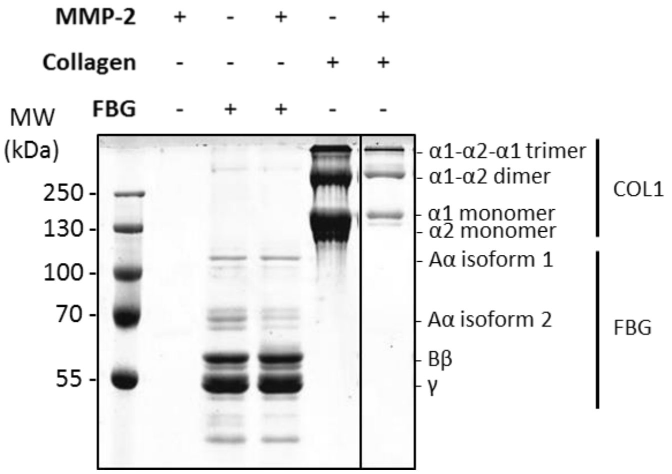

Figure S2: Cleavage of purified collagen type I and FBG by MMP-2. SDS-PAGE of fibrinogen (50 μg) and collagen type I (50 μg) degradation by recombinant MMP-2 (12 nM) after incubation for 24 hours at 37°C.

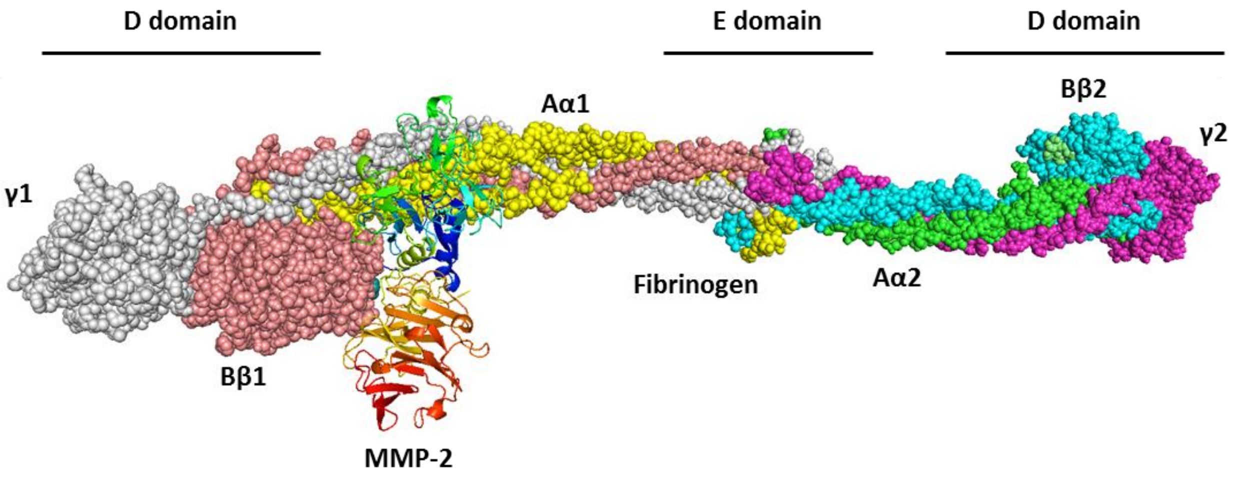

Fig. S3: Molecular docking of FBG and MMP-2. Whole annotated image of molecular interactions between MMP-2 and FBG presented in Fig. 7a.

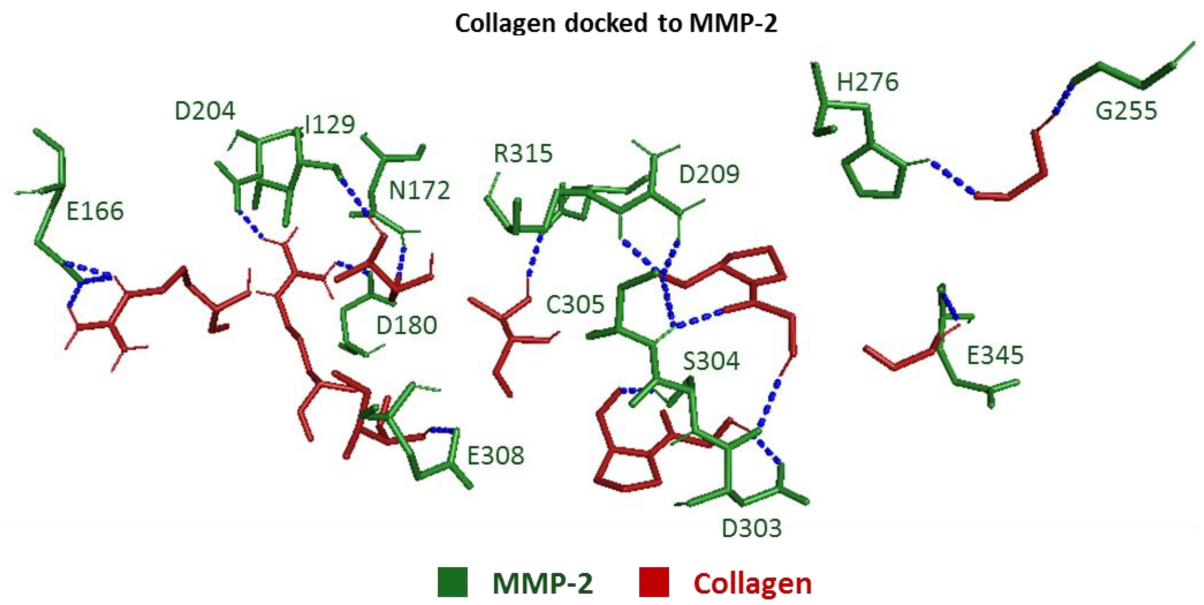

Figure S4: Molecular docking of collagen to MMP-2. Labelled residues of MMP-2 (green) that form hydrogen bonds (blue dotted lines) with collagen residues (red) are presented. Interacting MMP-2 residues span over the catalytic domain and the fibronectin type II repeats 1,2 and 3.

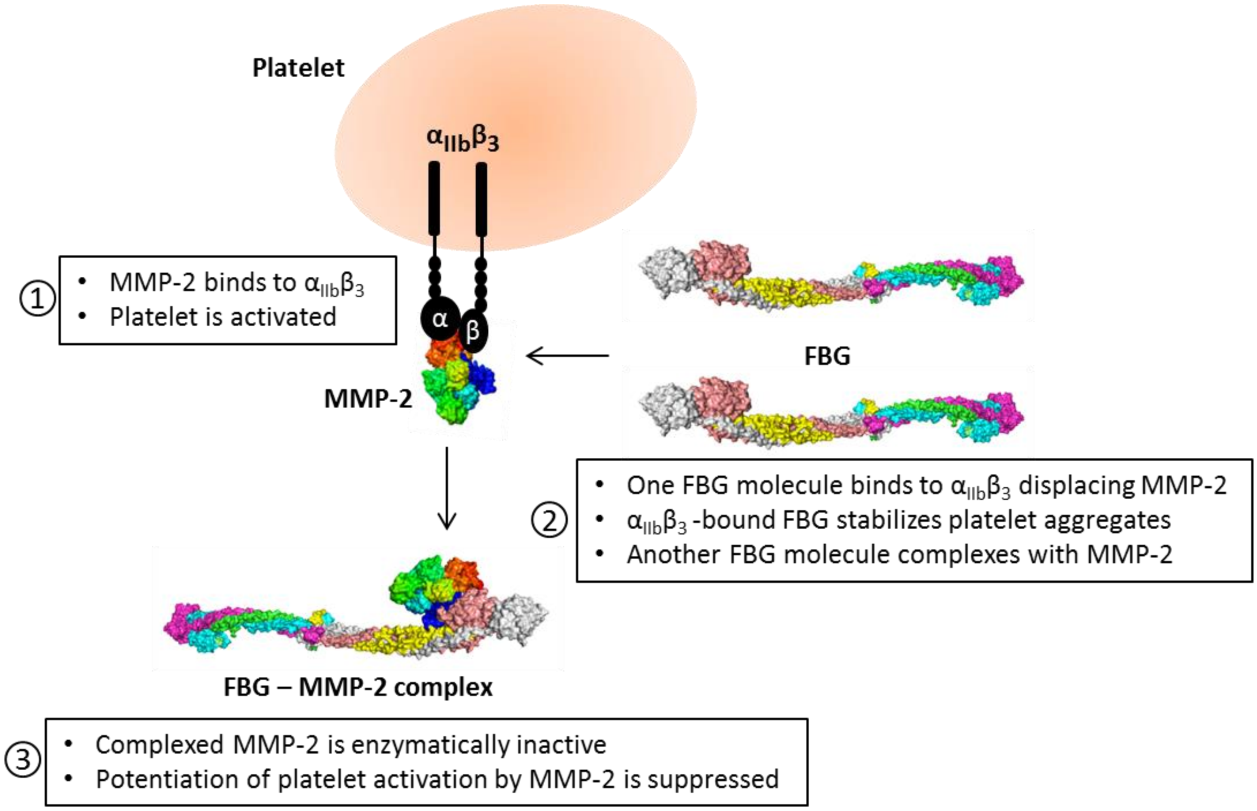

Figure S5: Illustration of a hypothesized anti-aggregatory function of FBG-mediated inhibition of MMP-2 activity.

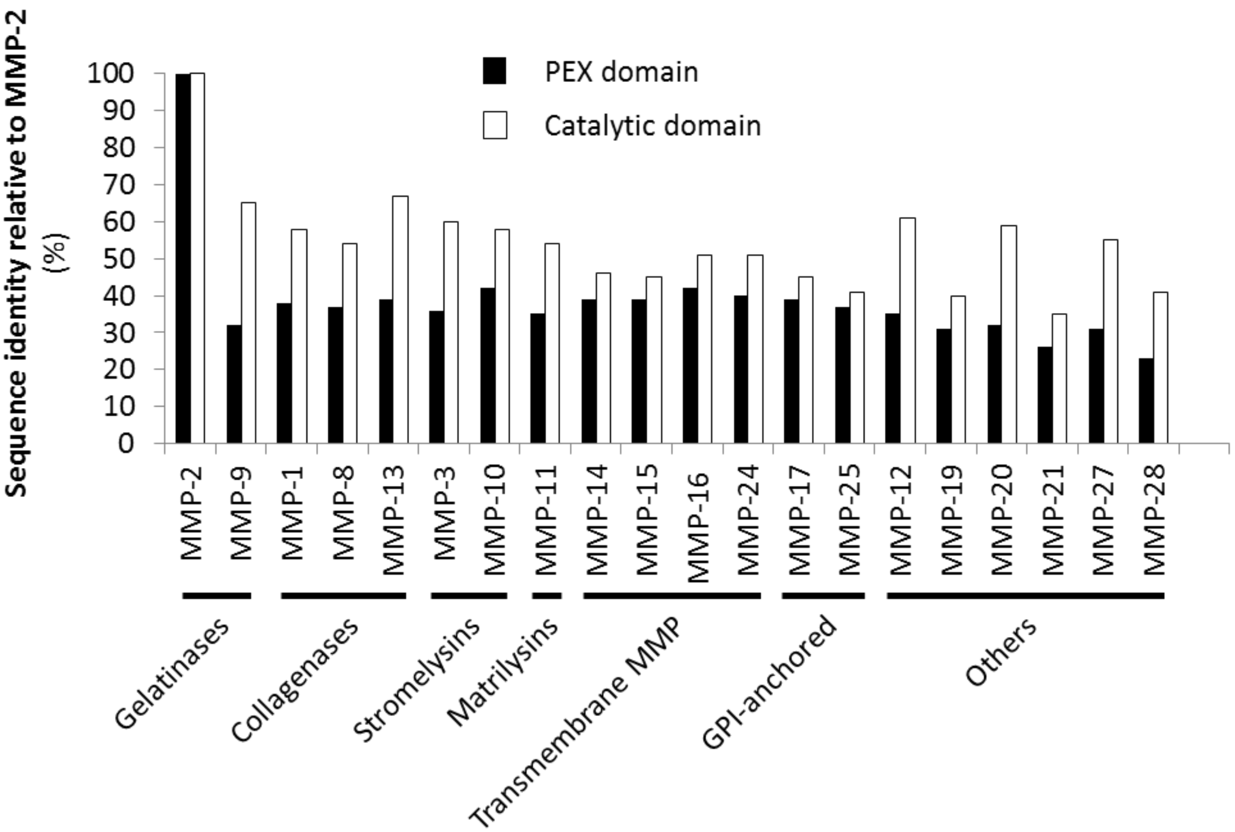

Figure S6: Comparison of amino acid sequence similarity of MMPs relative to MMP-2. The amino acid sequences of catalytic and the hemopexin-like domains of MMPs were aligned with MMP-2. The data is presented as percent identity relative to MMP-2.

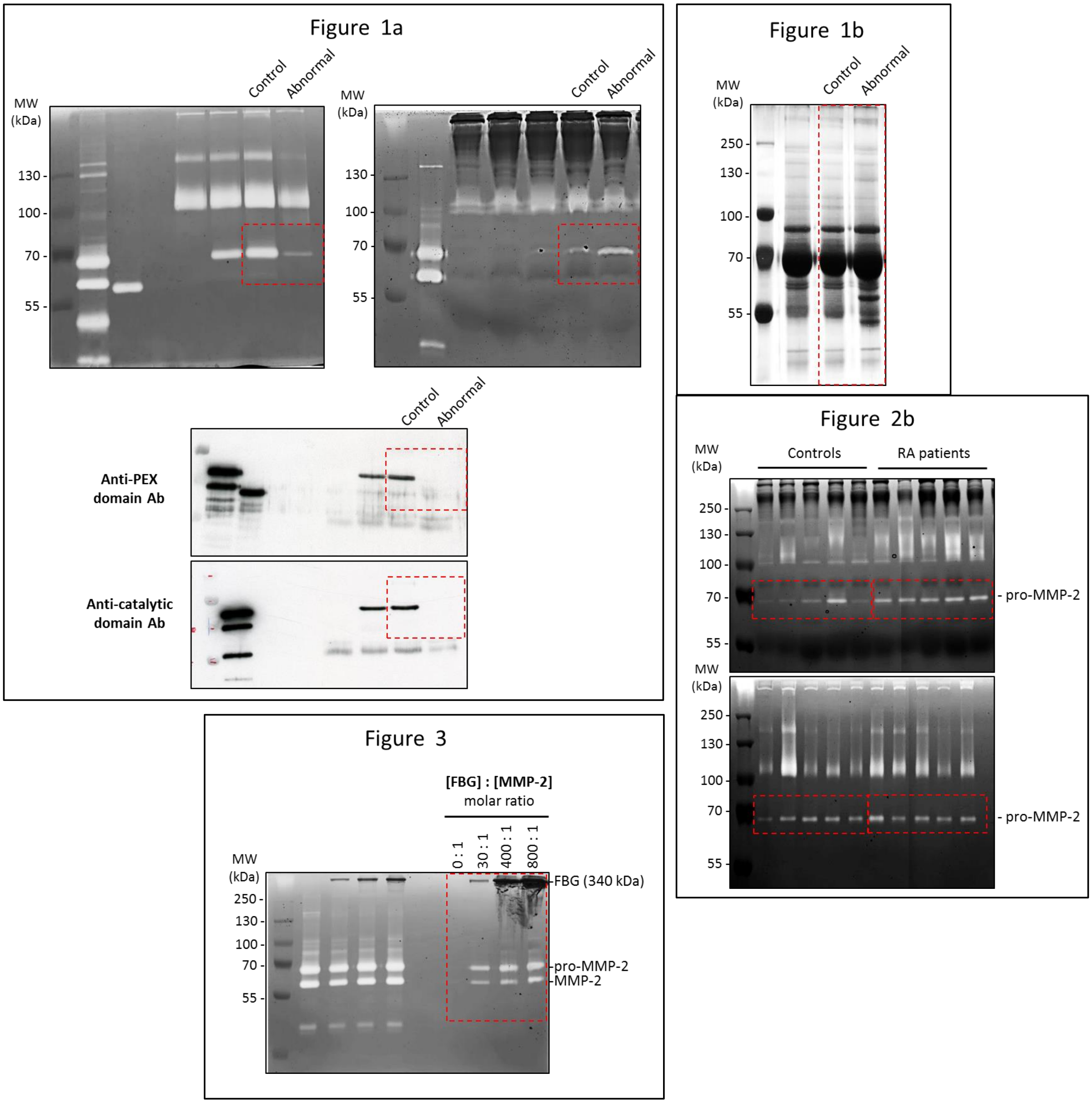

Figure S7: Full length gels and blots for figures 1a, 1b, 2b and 3.

Denotation	Condition	Age	Sex	Denotation	Condition	Age	Sex
C1 (abnormal)	Asymptomatic	30	M	R4	RA	70	F
C2	Control	69	F	R5	RA	76	F
C3	Control	41	M	R6	RA	36	F
C4	Control	69	F	R7	RA	64	M
C5	Control	60	M	R8	RA	68	F
C6	Control	77	F	R9	RA	35	F
C7	Control	44	M	R10	RA	42	F
C8	Control	35	F	R11	RA	40	M
C9	Control	43	F	R12	RA	69	F
C10	Control	59	F	R13	RA	45	F
C11	Control	42	F	R14	RA	34	F
C12	Control	53	F	R15	RA	56	F
C13	Control	34	F	R16	RA	66	M
C14	Control	37	F	R17	RA	57	F
C15	Control	65	F	R18	RA	40	F
C16	Control	71	M	R19	RA	36	F
C17	Control	36	F	R20	RA	66	F
C18	Control	69	F	R21	RA	69	F
C19	Control	45	F	R22	RA	74	F
C20	Control	67	F	R23	RA	44	M
R1	RA	53	F	R24	RA	58	F
R2	RA	58	M	R25	RA	47	F
R3	RA	38	F				

Table S1: **Demographic information of the total cohorts of sera donors assessed.**

	**Fibrinogen docked to MMP-2**	**Marimastat docked to MMP-2**
**Hydrogen bonds**	Y110, N111, E166, A167, E177, G181, Y182, P183, L190, A192, G200, D204, H407, E412, H413, S414, Q415, P423	H413, P423
**Other interacting residues**	F112, F113, P114, R115, R127, D164, G165, D168, I169, M170, H178, G179, D180, F184, D185, G189, L191, H193, V201, T311, I424, Y425	F184, G189, L190, A192, H193, A194, L399, V400, H403, H407, L420, A422, I424, Y425, T426
**Target domain(s) of MMP-2**	Catalytic<br>Fibronectin type II repeat 2	Catalytic

Table S2: A comparison of MMP-2 residues interacting (within 5Å) with FBG and Marimastat.

	**Collagen docked to MMP-2**
**Hydrogen bonds**	I129, E166, N172, D180, D204, D209, G255, H276, D303, S304, C305, E308, R315, E345
**Other interacting residues**	R127, G130, Y131, T132, P133, G165, M170, F173, H178, G200, V201, A278, C291, F293, S301, Y302, T306, T307, G309, R310, D312, G313, T335, S344, G346, A347, K386
**Target domain(s) of MMP-2**	Catalytic<br>Fibronectin type II repeat 1<br>Fibronectin type II repeat 2<br>Fibronectin type II repeat 3

Table S3: A comparison of MMP-2 residues interacting (within 5Å) with collagen in the presence or absence of FBG.
